# Supplementary material for: Bartonella quintana Infection in Canada: A Retrospective Laboratory Study and Systematic Review of the Literature
Source: Pathogens. 2024 Dec 6;13(12):1071. doi: 10.3390/pathogens13121071 (PMC11728599; doi:10.3390/pathogens13121071)
Supplement: Supplementary file 1 [file pathogens-13-01071-s001.zip › File S2.pdf]

Appendix 2. Description of data extracted from included publications

- Publication data: last name of study's first author, year of publication.
- Epidemiologic data: age and sex of the patient, location of likely *B. quintana* acquisition, history of houselessness.
- Clinical data: *B. quintana* syndrome (e.g., endocarditis, febrile illness, bacillary angiomatosis…), presence of embolization (for cases of endocarditis), treatment and clinical outcome (survival vs. death).
- Data relating to *B. quintana* diagnosis: molecular target of *B. quintana*, location of molecular testing, whether qPCR was performed at the NML and IFA titer result.
