# Supplementary material for: Bartonella quintana Infection in Canada: A Retrospective Laboratory Study and Systematic Review of the Literature
Source: Pathogens. 2024 Dec 6;13(12):1071. doi: 10.3390/pathogens13121071 (PMC11728599; doi:10.3390/pathogens13121071)
Supplement: Supplementary file 1 [file pathogens-13-01071-s001.zip › File S3.pdf]

Appendix 3. Four cases of *B. quintana* disease with paired tissue and whole blood samples and their associated cycle threshold values

**Case**	**1**	**2**	**3**	**4**
**Tissue Ct**	16.0 / 16.9	23.1 / 19.7	16.4 / 13.9	21.3 / 18.6
**Blood Ct**	37.1 / 36.5	38.4 / 37.5	39.3 / 35.1	35.3 / 32.3

Case: patient diagnosed with *B. quintana* disease by PCR performed at the NML. Ct: cycle threshold value. The first number indicates the cycle threshold value for the *Bartonella* screen using ITS3 gene targeting the *Bartonella* genus. The second number is the cycle threshold value of *yopP* gene targeting *B. quintana.*
